# Negative Events in Childhood Predict Trajectories of Internalizing Symptoms Up to Young Adulthood: An 18-Year Longitudinal Study

**DOI:** 10.1371/journal.pone.0114526

**Published:** 2014-12-08

**Authors:** Maria Melchior, Évelyne Touchette, Elena Prokofyeva, Aude Chollet, Eric Fombonne, Gulizar Elidemir, Cédric Galéra

**Affiliations:** 1 Inserm, UMR_S 1136, Pierre Louis Institute of Epidemiology and Public Health, Department of Social Epidemiology, F-75013, Paris, France; 2 Sorbonne Universités, UPMC Univ Paris 06, UMR-S 1136, Pierre Louis Institute of Epidemiology and Public Health, Department of Social Epidemiology, F-75013, Paris, France; 3 Groupe de Recherche en Inadaptation Psychosociale (GRIP), Laval University, School of Psychology, Québec City, Québec, Canada; 4 Brain Institute, Oregon Health & Science University, Portland, OR, United States of America; 5 Université de Bordeaux, Pôle Pédopsychiatrie Universitaire, Hôpital Charles-Perrens, INSERM U897, Bordeaux, France; United (Osaka U, Kanazawa U, Hamamatsu U Sch Med, Chiba U and Fukui U) Graduate School of Child Development, Japan

## Abstract

**Background:**

Common negative events can precipitate the onset of internalizing symptoms. We studied whether their occurrence in childhood is associated with mental health trajectories over the course of development.

**Methods:**

Using data from the TEMPO study, a French community-based cohort study of youths, we studied the association between negative events in 1991 (when participants were aged 4–16 years) and internalizing symptoms, assessed by the ASEBA family of instruments in 1991, 1999, and 2009 (n = 1503). Participants' trajectories of internalizing symptoms were estimated with semi-parametric regression methods (PROC TRAJ). Data were analyzed using multinomial regression models controlled for participants' sex, age, parental family status, socio-economic position, and parental history of depression.

**Results:**

Negative childhood events were associated with an increased likelihood of concurrent internalizing symptoms which sometimes persisted into adulthood (multivariate ORs associated with > = 3 negative events respectively: high and decreasing internalizing symptoms: 5.54, 95% CI: 3.20–9.58; persistently high internalizing symptoms: 8.94, 95% CI: 2.82–28.31). Specific negative events most strongly associated with youths' persistent internalizing symptoms included: school difficulties (multivariate OR: 5.31, 95% CI: 2.24–12.59), parental stress (multivariate OR: 4.69, 95% CI: 2.02–10.87), serious illness/health problems (multivariate OR: 4.13, 95% CI: 1.76–9.70), and social isolation (multivariate OR: 2.24, 95% CI: 1.00–5.08).

**Conclusions:**

Common negative events can contribute to the onset of children's lasting psychological difficulties.

## Introduction

Internalizing symptoms, defined as a range of emotional difficulties such as anxiety and depressive symptoms [1], [2] affect up to 15% of children and adolescents by the age of 16 [3], [4]. In 40% of cases, internalizing symptoms persist into adulthood and can lead to clinically significant psychopathology [2], [5]–[8] resulting in long-term health problems [9], difficulties in social adjustment [10], unemployment [11], and premature mortality [12].

Children who experience maltreatment, neglect [13]–[15], or sexual abuse [16], [17] are at high risk of having psychological problems including symptoms of depression and anxiety. Yet these severe negative events are infrequent, and a much higher burden of internalizing difficulties can be attributed to common negative events, such as high parenting stress [18] and parental divorce [19], [20]. However, to our knowledge, little is known about the long-term mental health consequences of specific negative childhood events.

Our study, based on the community-based French Trajectoires Epidémiologiques en Population (TEMPO) sample, examines the association between common negative events in childhood and trajectories of internalizing symptoms from childhood to young adulthood, adjusting for characteristics of individuals (sex, age) and their families (parental family status, socio-economic position, and parental history of depression) which can be associated with youths' internalizing symptoms [4], [21], [22].

## Materials and Methods

### Sample and Procedures

The TEMPO study sample has been described in detail elsewhere [23]. Briefly, the study was set up in 2009 among young adults aged 22–35 years, whose parents participate in the GAZEL cohort study (20,624 employees of a large French public-sector utility company followed since 1989) [24] and who took part in a study of children's mental health in 1991 and 1999 (the GAZEL Youth study). The original 1991 sample included 2,498 children aged 4–16 years, selected to match the main socio-demographic characteristics of children in France (number of children per family and occupational grade of head of household) [25]. In 2009, all living parents of children who took part in the GAZEL Youth Study in 1991 received a letter asking them to forward the TEMPO study questionnaire to their son/daughter. Between 1991 and 2009, 16 participants died and 4 were too ill or disabled to answer. The overall response rate to the TEMPO questionnaire was 44.5% (n = 1,103), which is comparable to response rates of other mental health surveys in France (Alonso et al., 2004). Leading reasons for non-participation were non-transmission of the questionnaire by the parent (34.4%) or the youth's lack of interest (28.5%). Compared to 2009 respondents, non-respondents were older, more likely to have parents who were divorced, and had low socioeconomic background but did not vary with regard to their parents or their own overall psychological characteristics.

In 1991, data on participating children were collected via parental reports. In 1999, data were collected via parental reports (n = 1,268) and youth self-reports (n = 1,148). In 2009, data were collected by youth self-reports (n = 1103). Factors associated with study participation at baseline and follow-up included younger age, non-divorced parents, and intermediate/high family socioeconomic background. The TEMPO study received approval from France's national committees for data protection (CCTIRS: Comité Consultatif sur le Traitement des Informations pour la Recherche en Santé and CNIL: Commission Nationale Informatique et Liberté).

### Measures

#### Negative childhood events

In 1991, parents were asked whether in the preceding 12 months their children had experienced the following negative events: 1) school difficulties, 2) parents under a lot of stress, 3) a serious illness/health problem, 4) social isolation, 5) the illness of a close family member/friend, 6) a family move, 7) parental divorce, 8) parental conflict, 9) death of a close family member/friend, 10) parental unemployment/financial problems, and 11) frequent parental absence from home. The number of negative childhood events was summed and studied as an ordinal variable (1, 2, and > = 3 vs.0).

#### Youths' internalizing symptoms

As described by Touchette et al., [2], youths' internalizing symptoms (that is symptoms of anxiety/depression, withdrawn behavior, psychosomatic complaints) were assessed in 1991, 1999 and 2009 using the ASEBA system [26]. This widely used instrument of 118 items assesses behaviour (internalizing and externalizing symptoms) over a six-month period and has previously been validated in French [27]–[29]. In 1991, participants' parents completed the Child Behaviour Checklist (CBCL); in 1999 parents and youths completed the Youth Self-Report (YSR); in 2009 youths completed the Youth Self-Report [30]. As advised by the ASEBA authors, items measuring youths' internalizing symptoms were ascertained by summing all relevant items (1991: n = 31, Cronbach's alpha: 0.83; 1999: n = 31, Cronbach's alpha: 0.88; 2009: n = 44, Cronbach's alpha: 0.93). To make these scales comparable, the three measures of internalizing symptoms were standardized: 1991: mean = 50.2, SD = 10.1, range = 37.6–101.2; 1999: 49.7, SD = 9.9, range = 34.9–90.6; 2009: mean = 50.0, SD = 10.1, range = 37.1–83.4.

#### Covariates

Unless indicated otherwise, covariates were measured at study baseline and included characteristics of youths: sex (male vs. female) and age (studied as a continuous variable, and>10 years old vs. < = 10 years old), and characteristics of their families: parental family status in 1991 (parents divorced/separated vs. two-parent family), family income (< = 1981 vs.>1981 €/month, which is roughly equivalent to average family income in France the same time period) [31], and parental history of depression, assessed through parental self-reports of depression in the yearly GAZEL study questionnaire (1989–2009) and TEMPO participants' reports of their parents' lifetime experience of depression on the National Institute of Health-Family Inventory for Genetic Studies (NIH-FIGS) questionnaire (yes vs. no) [32]. In additional analyses, we adjusted for participants' emotionality between ages 7 and 10 assessed retrospectively in 1999 using the EAS scale [33].

### Statistical analyses

As previously described [2], in order to identify participants' trajectories of internalizing symptoms between 1991 and 2009, we used semiparametric mixture models [34]. This yielded four distinct trajectory groups validated with the maximum Bayesian information criterion (BIC) (the BIC for 3-, 4-, and 5- group solutions were respectively: -12971.1, -12965.0 and -12967.4). 74.5% of participants (n = 1,119) had persistently low internalizing symptoms; 11.4% of participants (n = 171) had high internalizing symptoms in childhood which decreased during follow-up (high decreasing trajectory); 11.6% (n = 176) had low levels of internalizing symptoms in childhood which increased during follow-up (low increasing trajectory); and 2.5% of participants (n = 37) had persistently high internalizing symptoms. Each study participant was assigned to a specific trajectory based on a posterior probability of belonging to that group.

To test the association between participants' experience of negative childhood events (number and type of events) and internalizing symptoms trajectories, we first tested univariate associations using the chi-square statistic. Second, we used multinomial regression models to test associations between the number of negative events and all potential covariates (sex, age, parental family status, socio-economic position, and parental history of depression) and internalizing symptoms trajectories. All potential covariates associated with internalizing symptoms trajectories with a p-value of 0.05 were retained for the multivariate analyses. Third, we studied associations between the number of negative childhood events and internalizing symptoms trajectories in multivariate regression models adjusted for covariates. Fourth, we tested associations between specific negative childhood events and participants' internalizing symptoms trajectories adjusting for covariates. In additional analyses, we tested interactions between negative events and participants' sex and age. Moreover, restricting the sample to participants with complete 1991, 1999, and 2009 data (n = 674), we tested whether the association between negative childhood events and internalizing symptoms trajectories was not explained by high childhood emotionality.

Analyses were performed using SPSS (version 16.0, SPSS Inc, Chicago, ILL) and SAS V9 (SAS Institute, 2006).

## Results

Overall, the study included 54.6% of females and the mean age of study participants in 1991 was 10.4 years (SD = 3.64). Individuals who were older than 10 years of age were more likely to have high decreasing (OR = 1.59; 95% CI: 1.11–2.29) and less likely to have low increasing (OR = 0.49; 95% CI: 0.32–0.73) internalizing symptoms trajectories.

**Figure 1:**
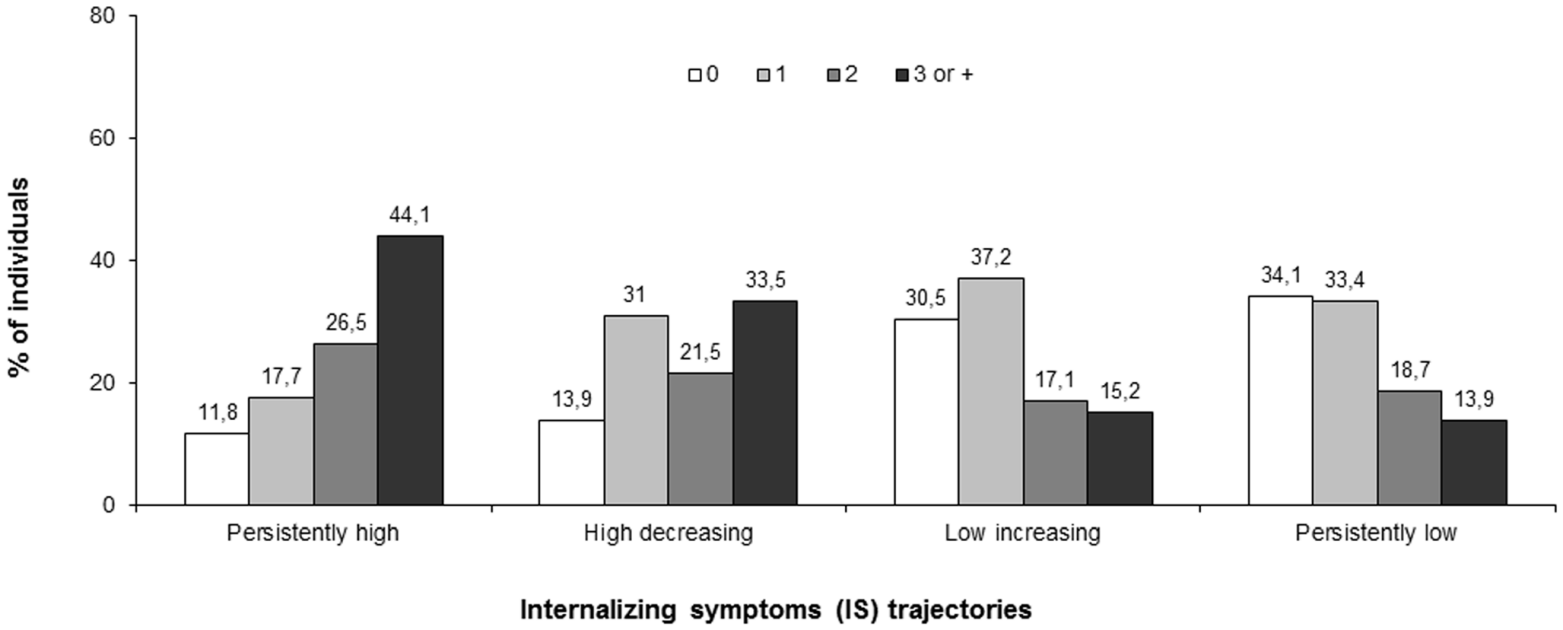
Number of childhood negative events and internalizing symptoms trajectories 1991–2009 (French TEMPO study, n = 1503, %).

As shown in **Fig. 1**, participants with 3 or more negative events in childhood were most likely to have persistently high (44.1%) or high decreasing (33.5%) internalizing symptoms.

**Table 1** presents age and sex-adjusted associations between negative events in childhood as well as potential covariates and participants' internalizing symptoms trajectories. Negative events in childhood were associated with the likelihood of experiencing persistently high internalizing symptoms (age and sex-adjusted ORs: one negative event: OR = 1.53, 95% CI: 0.43–5.49; two negative events: OR = 4.40, 95% CI: 1.33–14.55; three or more negative events: OR = 10.57, 95% CI: 3.42–32.70), as well as high decreasing symptoms (age and sex-adjusted ORs: one negative event: OR = 2.28, 95% CI: 1.35–3.86; two negative events: OR = 2.90, 95% CI: 1.65–5.11; three or more negative events: OR = 6.33, 95% CI: 3.70–10.84). Negative events in childhood were not associated with a low increasing internalizing symptoms trajectory.

**Table 1:** Negative childhood events and potential covariates associated with internalizing symptoms trajectories (French TEMPO study, 1991–2009, n = 1503, age and sex-adjusted ORs, 95% CI).

		Internalizing symptoms trajectories								
	Prevalence	Persistently high, n = 37 (2.5%)‖			High decreasing, n = 171 (11.4%)‖			Low increasing, n = 176 (11.6%)‖		
	n (%)	OR	95% CI	P	OR	95% CI	P	OR	95% CI	P
**Negative events (1991)**										
≥3 negative events	237 (17.0)	**10.57**	**(3.42**–**32.70)**	**<.0001**	**6.33**	**(3.70**–**10.84)**	**<.0001**	1.24	(0.73–2.09)	0.43
2 negative events	265 (19.0)	**4.40**	**(1.33**–**14.55)**	**0.02**	**2.90**	**(1.65**–**5.11)**	**0.0002**	1.06	(0.64–1.76)	0.81
1 negative event	463 (33.2)	1.53	(0.43–5.49)	0.51	2.28	(1.35–3.86)	**0.002**	1.25	(0.83–1.89)	0.28
**Potential covariates (1991),** †										
Sex, girls	821 (54.6)	**4.16**	**(1.81**–**9.55)**	**0.0008**	**1.39**	**(1.00**–**1.93)**	**0.05**	**2.50**	**(1.76**–**3.55)**	**<.0001**
Age at baseline (1991),>10 years old	733 (48.9)	1.46	(0.75–2.86)	0.27	1.45	(1.05–2.01)	**0.03**	0.51	(0.36–0.71)	**<.0001**
Parental divorce	115 (7.7)	0.70	(0.16–2.96)	0.62	1.48	(0.86–2.54)	0.15	1.00	(0.54–1.85)	0.99
Familial socio-economic position, <median family income (1.981 €/mo)	539 (37.0)	0.62	(0.30–1.32)	0.22	1.25	(0.90–1.75)	0.19	0.91	(0.65–1.29)	0.60
Parental history of depression (1989–2009)	387 (26.6)	**2.58**	**(1.31**–**5.11)**	**0.006**	**2.16**	**(1.52**–**3.05)**	**<.0001**	**1.53**	**(1.08**–**2.18)**	**0.02**

‖ compared with a trajectory of individuals who reported persistently low internalizing symptoms (n = 1119).

† Multinomial regression models were adjusted on sex and age at baseline.

Adjusting for all relevant covariates (**Table 2** **)**, ORs of internalizing symptoms associated with negative events in childhood somewhat decreased but remained elevated and statistically significant. Participants with negative events in childhood had an increased likelihood of experiencing persistently high internalizing symptoms (multivariate ORs: one negative event: OR = 1.51, 95% CI: 0.42–5.41; two negative events: OR = 4.14, 95% CI: 1.25–13.76; three or more negative events: OR = 8.94, 95% CI: 2.82–28.31) or high decreasing internalizing symptoms (multivariate ORs: one negative event: OR = 2.19, 95% CI: 1.29–3.72; two negative events: OR = 2.50, 95% CI: 1.40–4.46; three or more negative events: OR = 5.54, 95% CI: 3.20–9.58).

**Table 2:** Negative childhood events and internalizing symptoms trajectories (French TEMPO study, 1991–2009, n = 1503, multivariate ORs, adjusted for sex, age at baseline and parental depression, 95% CI).

		Internalizing symptoms trajectory (IS)								
	Prevalence	Persistently high, n = 37 (2.5%)^‖^			High decreasing, n = 171 (11.4%)^‖^			Low increasing, n = 176 (11.6%)^‖^		
*	n (%)	OR	95% CI	*P*	OR	95% CI	*P*	OR	95% CI	*P*
Negative events in childhood										
≥3 negative events	237 (17.0)	**8.94**	**(2.82**–**28.31)**	**0.02**	**5.54**	**(3.20**–**9.58)**	**<.0001**	1.06	(0.61–1.84)	0.84
2 negative events	265 (19.0)	4.14	(1.25–13.76)	0.07	2.50	(1.40–4.46)	**0.002**	0.98	(0.59–1.64)	0.94
1 negative event	463 (33.2)	1.51	(0.42–5.41)	0.60	2.19	(1.29–3.72)	**0.004**	1.25	(0.83–1.88)	0.29
Sex, girls	821 (54.6)	**5.75**	**(2.18**–**15.16)**	**0.0004**	**1.50**	**(1.05**–**2.14)**	**0.03**	**2.28**	**(1.58**–**3.28)**	**<.0001**
Age at baseline (1991),>10 years old	733 (48.9)	1.68	(0.81–3.48)	0.17	**1.43**	**(1.00**–**2.05)**	**0.05**	0.53	(0.37–0.76)	**0.0005**
Parental history of depression (1989–2009)	387 (26.6)	1.68	(0.80–3.53)	0.17	**1.73**	**(1.19**–**2.52)**	**0.004**	**1.58**	**(1.09**–**2.29)**	**0.02**

Compared with a trajectory of individuals who reported persistently low internalizing symptoms (n = 1119).

Testing the role of specific negative events (**Table 3**), we observed that experiences most strongly associated with persistently high internalizing symptoms were school difficulties (multivariate OR: 5.31, 95% CI: 2.24–12.59), parental stress (multivariate OR:4.69, 95% CI: 2.02–10.87), childhood illness/serious health problem (multivariate OR: 4.13, 95% CI:1.76–9.70), and social isolation (multivariate OR: 2.24, 95% CI: 1.00–5.08). The same specific negative events were associated with a high decreasing trajectory, but the associated ORs were somewhat lower.

**Table 3:** Specific negative events in childhood and internalizing symptoms trajectories (French TEMPO study, 1991–2009, n = 1503, multivariate ORs adjusted for sex, age at baseline, and parental history of depression, 95% CI).

		Internalizing symptoms (IS) trajectories								
	Prevalence	Persistently high, n = 37 (2.5%)‖			High decreasing, n = 171 (11.4%)‖			Low increasing, n = 176 (11.6%)‖		
**Negative childhood events**	n (%)	OR¶	95% CI	*P*	OR¶	95% CI	*P*	OR¶	95% CI	*P*
School difficulties	199 (13.6)	**5.31**	**(2.24**–**12.59)**	**0.0002**	**3.57**	**(2.31**–**5.50)**	**<.0001**	1.18	(0.67–2.07)	0.56
Parents under a lot of stress	263 (17.9)	**4.69**	**(2.02**–**10.87)**	**0.0003**	**1.86**	**(1.16**–**2.96)**	**0.01**	1.38	(0.84–2.25)	0.20
Illness/serious health problem	171 (11.6)	**4.13**	**(1.76**–**9.70)**	**0.001**	**2.12**	**(1.29**–**3.48)**	**0.003**	1.09	(0.62–1.94)	0.76
Social isolation	306 (20.5)	**2.24**	**(1.00**–**5.08)**	**0.05**	**2.02**	**(1.35**–**3.02)**	**0.0006**	0.97	(0.62–1.51)	0.88
Illness of a close family member/friend	404 (27.5)	0.93	(0.41–2.10)	0.86	0.96	(0.64–1.43)	0.83	0.67	(0.44–1.02)	0.06
Family move	103 (7.0)	1.35	(0.36–5.08)	0.66	0.48	(0.20–1.20)	0.12	1.74	(0.96–3.16)	0.07
Parental divorce	115 (7.7)	0.20	(0.03–1.65)	0.14	1.02	(0.54–1.92)	0.96	0.93	(0.47–1.85)	0.84
Parental conflict	101 (6.9)	1.16	(0.38–3.52)	0.80	1.15	(0.60–2.19)	0.68	0.70	(0.31–1.61)	0.40
Death of a close family member/friend	48 (3.3)	0.85	(0.10–7.40)	0.88	1.19	(0.46–3.05)	0.72	1.16	(0.43–3.11)	0.77
Parental unemployment/financial problems	108 (7.3)	1.75	(0.59–5.18)	0.32	1.65	(0.90–3.03)	0.11	1.04	(0.50–2.17)	0.91
Frequent parental absence from home	208 (14.2)	1.52	(0.62–3.74)	0.36	1.06	(0.64–1.77)	0.81	0.87	(0.50–1.49)	0.61

‖ compared with a trajectory of individuals who reported persistently low internalizing symptoms (n = 1119).

¶ Associations between negative events and internalizing symptoms trajectories were tested in multinomial regression models adjusted for all specific negative childhood events and all covariates.

We found no statistically significant interactions between negative childhood events and participants' sex and age. Nonetheless, the association between negative childhood events and a persistently high internalizing symptoms trajectory was somewhat higher in participants who were younger (multivariate OR among participants younger than 10 years of age at study baseline: 10.75, 95% CI 1.24–93.05 vs. 8.04, 95% CI 1.96–32.89 in participants 10 or older at study baseline). In additional analyses restricted to a subsample of our study population, the association between three or more negative events and the likelihood of experiencing persistently high or high decreasing symptoms was additionally decreased after adjustment for high emotionality, but remained elevated and statistically significant (multivariate ORs respectively: 5.84, 95% CI 1.26–27.13 and 6.74, 95% CI 2.82–16.09), implying that the association between negative childhood events and internalizing symptoms is not explained by preexisting temperament.

## Discussion

### Main findings

Our study, based on a community-based sample of youths, indicates that the experience of negative events in childhood is associated with high levels of internalizing symptoms that sometimes persist into adulthood. Specifically, after adjusting for all covariates, compared to youths who did not experience negative life events, those with three or more negative events were 8.9 times more likely to have persistently high internalizing symptoms and 5.5 times more likely to have high levels of internalizing symptoms in childhood that later decreased. The probability of high internalizing symptoms was most strongly associated with youths' school difficulties, parental stress, childhood illness/serious health problem, and social isolation.

### Limitations and strengths

Our study has limitations, which need to be acknowledged before we interpret our findings. First, we did not consider severe events such as maltreatment or sexual abuse, which can be associated with children's internalizing symptoms [13], [16]. However, such severe occurrences are rare – in France, approximately 8% of young adults report ever experiencing physical violence and less than 1% sexual abuse [35]– and therefore only account for a small proportion of cases of depression and anxiety in the population. Moreover, child neglect and maltreatment may occur in the context of family dysfunction, which we accounted for by controlling for parental separation and parental stress [36]. Still, common negative life events may co-occur with more severe forms of neglect and future research should examine both types of negative childhood experiences on children's mental health. Second, some of the negative events we considered, such as school difficulties and social isolation can be chronic and result from the child's emotional difficulties rather than cause them. Nonetheless, evidence indicating that negative events and emotional symptoms mutually reinforce each other and that the direction of the causal association is difficult to ascertain imply that life experiences can contribute to emotional symptoms (Kim et al., 2003). TEMPO study participants were on average 10 years old at the time of the baseline assessment and it is reasonable to assume that negative events preceded the occurrence of internalizing symptoms in most cases. Third, negative events and children's initial internalizing symptoms were ascertained by parents, which could introduce report bias. Nonetheless, at young ages, parents' reports of youths behavioral and emotional difficulties appear to be more accurate than children's own assessments, implying that the measures we used successfully captured participants' psychological problems early on [37]. In adolescence, we combined parental and youths' reports, thereby increasing the validity of our assessment. Fourth, we studied youths who had at least one parent who was an employee of a large public-sector utility company, that is with stable employment. In the general population, the occurrence of certain negative events could be more frequent than we report.

Our study also has strengths. First, we studied the association between childhood negative events and trajectories of internalizing symptoms over a period of 18 years in a sample of community-based youths. Second, we studied the role of common negative events, which are likely to contribute to a greater number of cases of anxiety and depression in the population than more severe but rare experiences such as maltreatment. Third, we used ASEBA scores, which are less specific than psychiatric diagnoses but have satisfactory psychometric properties and have frequently been used to screen for clinically significant depressive/anxiety problems throughout the life course [38].

### Childhood negative events and internalizing symptoms trajectories

In our study, participants' likelihood of experiencing high levels of internalizing symptoms, which sometimes persisted over time, was associated with the number of negative events. Interestingly, associations between negative events and persistently high vs. high decreasing internalizing symptoms were comparable, suggesting that negative events primarily play a role in the onset of psychological difficulties. These findings are consistent with the kindling hypothesis, which postulates that negative events play a role in the onset of an initial episode of depression but less so in relation to later episodes [39], [40]. According to this model and the stress sensitization theory, in individuals with a high sensitivity to stress (for example those with a genetic predisposition or an unstable, highly emotional, temperament) even minor negative events can trigger the onset of depression [39]. Similarly, negative childhood events can contribute to the onset of symptoms of anxiety [41], [42]. Our results are consistent with the kindling hypothesis of the origins of internalizing symptoms early on in life.

Among negative childhood events which we studied, school difficulties, parental stress, childhood illness, and social isolation appeared most strongly associated with persistent high and high decreasing trajectories of internalizing symptoms. All of these experiences are perceived as stressful by children and their parents, which could explain their association with internalising symptoms [43]. Additionally, school difficulties and social isolation could also partly reflect a temperamental predisposition to psychological difficulties – either through externalizing behaviors, inattention and lack of concentration [44], [45] or inhibition and high emotionality [46], [47]. Finally, parental stress may also co-occur with parents' symptoms of depression and anxiety which increase the likelihood of harsh and overprotective parenting, and could also exert an influence on children's' psychological well-being [48]–[51].

Consistent with prior research, we found that girls and children raised by parents who had a history of depression had a high likelihood of internalizing symptoms, which sometimes persisted over time [43], [52]–[56]. These results highlight the long-term importance of early life characteristics as well as familial factors with regard to children's mental health [57].

## Conclusion

Overall, our study shows that common negative events in childhood are associated with high internalizing symptoms in childhood, which sometimes persist later in the life. This effect appears to be similar across sexes and age groups, and depends on the number as well as the type of negative events experienced. Parents and mental health specialists should be aware that common negative events can predict a negative cycle of emotional disturbances which can last up until adulthood.
